# Anaemia prevalence and dietary diversity among women in the rural Free State, South Africa

**DOI:** 10.4102/hsag.v25i0.1421

**Published:** 2020-10-06

**Authors:** Elizabeth M. Jordaan, Violet L. van den Berg, Francois C. van Rooyen, Corinna M. Walsh

**Affiliations:** 1 Department of Nutrition and Dietetics, Faculty of Health Sciences, University of the Free State, Bloemfontein, South Africa; 2 Department of Biostatistics, Faculty of Health Sciences, University of the Free State, Bloemfontein, South Africa

**Keywords:** anaemia, dietary diversity, folate, iron, iron deficiency

## Abstract

**Background:**

Anaemia, a global public health problem that particularly affects women, holds major consequences for human health.

**Aim:**

Determining dietary diversity, prevalence of anaemia and contraception use.

**Setting:**

Rural women, 25–49 years, in the Free State Province, South Africa.

**Methods:**

In a cross-sectional descriptive quantitative study, dietary diversity was determined with a 24-h recall; biochemical markers of anaemia, iron deficiency and inflammation were measured; and contraceptive use was recorded.

**Results:**

Of 134 women (median age 41 years), 51.5% had medium, and 44.8% had low dietary diversity. Overall, 76.9% consumed flesh meats and fish, but only 25.4% ate dark green leafy vegetables. Anaemia was present in 4.6%; 1.5% presented with iron deficiency; and 0.7% presented with iron deficiency anaemia, evidenced by low ferritin levels. However, 45.0% had elevated C-reactive protein (CRP). Overall, 7.5% presented with elevated homocysteine levels, but only 3.8% had low red cell folate levels. More than half (54.1%) reported menstruating regularly and 71.6% used injectable contraceptives. Significant associations were found between median mean corpuscular volume (MCV) and mean corpuscular haemoglobin (MCH) and dietary diversity score.

**Conclusions:**

Although the prevalence of anaemia is low in this population, elevated CRP in almost half indicates that inflammation may mask iron deficiency. The older median age of the sample and approximately half of the women not menstruating regularly may also contribute to the low anaemia prevalence. Attention should be given to the women’s diets as almost half consume diets of low diversity, and not all consume foods rich in haemopoietic nutrients.

## Introduction

The global public health problem of anaemia affects women living in both developed and developing countries and holds major consequences for human health as well as economic development (Kassebaum & the Global Burden of Anemia Collaborators [GBD] 2016; World Health Organization [WHO] 2008). Although anaemia can occur in all stages of the lifecycle, it is more prevalent among women and young children (WHO 2014). Women of childbearing age lose iron through their menstrual blood loss, while pregnant women have an increased need for iron and therefore have a greater risk for developing anaemia (Milman 2011). According to findings of the South African National Health and Nutrition Examination Survey (SANHANES-1) published in 2013, the prevalence of anaemia among women of child-bearing age (15–54 years) ranged from 23.1% to 24.7% (Shisana et al. 2013). Research directed at nutritional anaemias largely focuses on iron-deficiency anaemia which remains the most dominant form of anaemia in the world (Kassebaum & GBD 2016). In 2011, 29% of non-pregnant women suffered from anaemia, while globally half a billion women of reproductive age are affected by it (Stevens et al. 2013). In contrast, however, little global data exist on the contribution of folate deficiency towards the development of anaemia (Balarajan et al. 2011).

Anaemia is defined as a significant reduction in the mass of circulating red blood cells, resulting in a diminished oxygen binding capacity of blood (Bunn 2011). Nutritional anaemias result from the insufficient bioavailability of haemopoietic nutrients (iron, vitamin B12 and folic acid) which could lead to symptoms including extreme fatigue and lethargy, amongst others (Biesalski & Erhardt 2007). A study conducted by Abrahams, Mchiza and Steyn (2011) concluded that a large proportion of the South African population is in a stage of nutrition transition where changes in dietary patterns are affecting health outcomes. This transition could also impact on anaemia.

The World Health Assembly aims to achieve a 50% reduction in the prevalence of anaemia amongst reproductive women by 2025 (WHO 2014). In order to improve maternal health and thus ensure a woman’s safe passage into motherhood, quality reproductive health services, accompanied by a series of well-timed interventions, should be implemented (UN 2010). A diet sufficient in all the required nutrients before and during pregnancy in order to enhance fertility, to support the pregnancy and the growing foetus, and to promote long-term health is of utmost importance (Derbyshire 2011), as the effect of poor nutritional status may follow both the mother and child for decades (Gallagher 2012).

Contraception could possibly play a role in lowering the risk for iron deficiency anaemia as it aids in reducing the number of pregnancies and the time interval between consecutive pregnancies (thus reducing the amount of iron needed for more pregnancies in a shorter time interval) (UNDP/UNFPA/WHO/WB 1998). The continuous use of contraceptives has been found to decrease the risk for anaemia significantly (Bellizzi & Ali 2018; Haile et al. 2017) possibly by reducing the amount of blood (menstrual) lost during this time (Yeasmin et al. 2010).

According to the WHO (2008), estimates of the prevalence of anaemia are only useful when associated with the prevailing causal factors (e.g. poor dietary intake) in specified settings. It is essential to collect accurate information concerning these factors, as they are multiple and complex and can serve as the basis for the development of appropriate interventions (WHO 2008).

Although various studies related to anaemia have been conducted in South Africa, limited data are available regarding the prevalence of anaemia among Southern African women in general and in the Free State province in particular. This study aimed to determine the prevalence of anaemia, dietary diversity, as well as contraceptive use in women aged 25–49 years in three rural towns in the Free State and to determine associations between biochemical indices and dietary diversity.

## Research methods and design

### Background

This study reports on findings from the ‘Assuring Health for All in the Free State’ (AHA-FS) research programme, which was an epidemiological study that aimed to determine how living in rural and urban areas affected lifestyle and indicators of health. The Free State Rural Development Partnership Programme (FSRDPP) includes the Trompsburg, Philippolis and Springfontein municipalities, where students from the Department of Nutrition and Dietetics at the University of the Free State do their rural internship. These three towns formed part of the rural phase of the AHA-FS study. A multidisciplinary research team investigated the socio-demographic status, household food security, dietary intake, levels of physical activity, as well as the knowledge, attitudes and practices related to nutrition, and reported health status of the study population by using standardised questionnaires. In addition to a medical examination, anthropometric measurements and blood specimens were also obtained for various investigations.

### Research design and aim

For the purpose of this study, data from the rural phase of the original study were used and applied in a cross-sectional investigation.

Haematological abnormalities of both HIV-infected and HIV-uninfected persons in the AHA study population have been published by Groenewald et al. (2011), but data on the risk factors associated with the development of anaemia within these communities have not been investigated.

### Population and sampling

All households in the township areas (excluding farms) in the rural towns of Trompsburg, Philippolis and Springfontein could participate in the original study. For this study, the total population of 134 women aged 25–49 years who were HIV-uninfected, gave informed consent and were not pregnant at the time of data collection were selected from the AHA-FS database.

### Measuring instruments and procedures

Information was obtained via structured interviews. A 24-h recall was used to determine individual dietary diversity. The dietary diversity score was determined by using the tool developed by the Food and Agriculture Organization (FAO). This tool summarises the number of food groups obtained from the 24-h recall into nine standardised food groups, namely starchy staples, dark green leafy vegetables, other vitamin A-rich fruit and vegetables, other fruits and vegetables, organ meat, flesh meat and fish, eggs, legumes, nuts and seeds and milk and milk products (FAO 2011). According to this tool, a low dietary diversity score was allocated if three food groups or less were consumed, a medium score where four to five food groups were consumed and a high score where six or more food groups were consumed. Questions relating to contraceptive use were included in the health questionnaire.

After an overnight fast, staff and registrars from the Department of Chemical Pathology, University of the Free State, collected respondents’ blood the next morning. The samples were stored immediately in ice-filled containers and transported to the laboratory. The blood samples were analysed according to standard techniques at the National Health Laboratory Service Laboratory at the University of the Free State. Full blood counts were determined using a Roche Sysmex XT 2000i® analyser, serum ferritin using a Beckman Coulter Synchron LX20, and transferrin and homocysteine levels using BN Prospec System nephelometric technology. Serum folate levels were recorded using a Bayer Advia Centaur System. C-reactive protein (CRP) was determined by means of the immunoturbidimetric method.

Anaemia was diagnosed when haemoglobin levels were below 12.0 g/dL (WHO 2008). According to the WHO (2008), iron deficiency is diagnosed when ferritin levels are below 15.0 ng/mL and haemoglobin levels are still greater than 12.0 g/dL. Iron deficiency anaemia is diagnosed when both ferritin and haemoglobin levels are below 15.0 ng/mL and 12.0 g/dL, respectively. These cut-off values were also used in this study to define iron deficiency and iron deficiency anaemia. CRP was considered elevated if greater or equal to 5 mg/L (NHLS reference values).

### Statistical analysis

Statistical analysis was performed using the Predictive Analytics SoftWare (PASW) Statistics Student version 22.0 by Statistical Package for the Social Sciences (SPSS) (IBM 2013). Frequencies and percentages were recorded for categorical data and medians and percentiles for continuous data. Only six out of the 134 women in the sample suffered from anaemia (haemoglobin < 12.0 g/dL), which made determining associations, especially between women suffering from anaemia and those who did not, difficult. The Kruskal–Wallis test and post-hoc Dunn’s test were used to compare iron and anaemia status markers among dietary diversity scores. Type I error rate was set at 5% (*p* < 0.05).

### Ethical considerations

Ethical approval was obtained from the Health Sciences Research Ethics Committee of the University of the Free State (ETOVS number 21/07), the Department of Health and local municipalities, 2007/01/25. Each respondent was assigned a particular number to ensure confidentiality. Ethical approval was obtained from the Health Sciences Research Ethics Committee of the University of the Free State the Department of Health and local municipalities. Participation was voluntary and respondents could withdraw from the study at any time.

## Results

Of the complete data set available as part of the “AHA-FS” study, 38.5% related to women between 25 and 49 years of age. After applying the exclusion criteria, 23.3% of the original data set could be included in the current study, resulting in a sample size of 134 women, with a median age of 41 years.

The women’s diet was predominantly based on starchy staples (Table 1). Flesh meat and fish, which are rich sources of iron, were consumed by more than three-quarters of the women, whereas sources of folate (dark green leafy vegetables and organ meats) were consumed by only approximately a quarter of the sample.

**TABLE 1:** Women’s consumption of food groups (*n* = 134).

Food group	*n*	%
Starchy staples	134	100
Dark green leafy vegetables	34	25.4
Other vitamin A-rich fruit and vegetables	15	11.2
Other fruits and vegetables	78	58.2
Organ meat	2	1.5
Flesh meat and fish	103	76.9
Eggs	14	10.4
Legumes, nuts and seeds	11	8.2
Milk and milk products	109	81.3

Overall, almost half (44.8%) of the women had a low dietary diversity score, while 51.5% had a medium dietary diversity score. Only a relatively small percentage (3.7%, 5/134) of the women had a high dietary diversity score (Table 2).

**TABLE 2:** Dietary diversity scores among the women (*n* = 134).

Dietary diversity score	*n*	%
Low (≤ 3 food groups consumed per day)	60	44.8
Medium (4–5 food groups consumed per day)	69	51.5
High (≥ 6 food groups consumed per day)	5	3.7

The percentage of women in this study presenting with low dietary diversity scores (≤ 3 food groups consumed per day) differs from various other studies related to dietary diversity undertaken in South Africa (Table 3).

**TABLE 3:** Low dietary diversity scores compared with other South African studies.

Study	% of sample
**Current study**	
Women aged 25–49 years	44.8
**Chakona and Shackleton (2017)**	
*Women in Richards Bay, Dundee and Harrismith, South Africa*	
Urban	69.0
Peri-urban	81.0
Rural	83.0
**SANHANES-1 (Shisana et al. 2013)**	
Men and women in the Free State (> 15 years)	45.1
**Oldewage-Theron and Kruger (2011)**	
Women in a peri-urban informal settlement in South Africa	80.9
**Labadarios, Steyn and Nel (2011)**	
Adult men and women in the Free State	26.6

The medians for all the blood values (haemoglobin, haematocrit, MCV, MCH, transferrin saturation, ferritin, homocysteine, red cell folate levels and CRP) were within normal limits (Table 4).

**TABLE 4:** Markers of anaemia and micronutrient status among women aged 25–49 years.

Blood samples	Normal reference value†,‡	*n*	Median (25th, 75th)	Low (%)	Normal (%)	High (%)
Haemoglobin	> 12.0 g/dL	130	13.8 (13.3, 14.5)	4.6	95.4	–
Haematocrit	> 0.371 L/L	131	0.429 (0.409, 0.446)	3.1	96.9	–
MCV	79.1–98.9 fl	131	93.8 (89.7, 98.4)	3.1	77.1	19.8
MCH	27.0–32.0 pg/cell	131	30.4 (28.8, 32.2)	7.6	67.2	25.2
Transferrin saturation	> 15%	74	26.1 (18.9, 34.8)	12.2	87.8	–
Ferritin	6–120 ng/mL	74	94.0 (48.5, 180.0)	1.4	58.1	40.5
Homocysteine	2.10–15.70 μmol/L	134	13.8 (13.3, 14.5)	–	92.5	7.5
Red cell folate	> 372 nmol/L	131	575.3 (487.8, 674.7)	3.8	96.2	–
CRP	5 mg/dL	120	4.6 (1.4, 8.0)	–	55.0	45.0

MCV, mean corpuscular volume; MCH, mean corpuscular haemoglobin; CRP, C-reactive protein.

† , Ampath 2010. Ampath desk reference: guide to laboratory tests. Centurion: Ampath.

‡ , National Health Laboratory Services Reference values. 2014.

Low haemoglobin levels were observed in 4.6% (6/134) of the women with only a small percentage presenting with low haematocrit (3.1%, 4/134), mean corpuscular volume (MCV) (3.1%, 4/134) and mean corpuscular haemoglobin (MCH) (7.6%, 10/134). Elevated MCH was present in a quarter (25.2%, 33/134) of the women.

Below normal transferrin saturation was found in 12.2% (9/134) of the women, with 1.4% (1/134) displaying low ferritin levels. The prevalence of anaemia, iron deficiency and iron deficiency anaemia in the current study was much lower than the findings of the SANHANES-1 published in 2013 (Table 5). Even though only 4.6% of the sample suffered from anaemia, seven out of the 134 women (5.2%) who presented with a low MCH, but normal haemoglobin levels were at risk for developing anaemia.

**TABLE 5:** Prevalence of anaemia and iron deficiency anaemia in comparison to SANHANES-1 findings.

Study	Anaemia (%)	Iron deficiency (%)	Iron deficiency anaemia (%)
**Current study**			
Women aged 25–49 years	4.6	1.5	0.7
**SANHANES-1 (Shisana et al. 2013)**			
South African women	23.1 (16–35 years)	5.9 (16–35 years)	9.7 (16–35 years)
	23.1 (35–44 years)	-	-
Women living in the Free State Province	17.6 (16–35 years)	2.0 (16–35 years)	8.8 (16–35 years)

Both the medians for homocysteine and red cell folate were also within normal limits. Elevated homocysteine levels occurred in 7.5% (10/134) of the women and 3.8% (5/134) had low red cell folate levels.

The median for CRP was normal (4.6 mg/L); however, 45.0% of the women presented with elevated CRP.

When assessing the prevalence of iron deficiency, it is important to consider menstruation patterns and contraceptive use (Table 6). Just over half (54.1%) of the women regularly menstruated and almost three quarters (71.6%) of them currently or previously made use of injectable contraceptives.

**TABLE 6:** Menstruation patterns and contraceptive use.

Questions	*n*	Yes		No	
		*n*	%	*n*	%
Still menstruated regularly	133	72	54.1	62	45.9
Currently or previously used injectable contraceptives	134	96	71.6	38	28.4

As expected, a significant association was found between median haemoglobin and whether the women still menstruated or not (*p* = 0.008) (Table 7). These median haemoglobin levels were within the normal range for both women who menstruated (13.7 g/dL) and women who did not (14.2 g/dL). No significant association was found between haemoglobin levels and women who currently or previously used injectable contraceptives and those that did not (*p* = 0.847). A significant association was found between ferritin levels and whether women had elevated CRP (*p* = 0.044).

**TABLE 7:** Statistically significant associations between blood parameters and other variables.

Variables	*n*	Median blood value (25th, 75th)	*p*-value for median differences
**Median haemoglobin levels across categories of menstruation:**			
Menstruated regularly	70	13.7 g/dL (13.0, 14.4)	0.008
Did not menstruate regularly	59	14.2 g/dL (13.5, 14.6)	
**Median ferritin levels across categories of CRP:**			
CRP elevated	39	120.0 ng/mL (60.0, 178.0)	0.044
CRP normal	27	62.0 ng/mL (32.0, 122.0)	
**Median MCV levels across categories of dietary diversity score:**			
Low dietary diversity	58	94.9 fl (91.1, 99.9)	0.029
Medium to high dietary diversity	73	92.9 fl (87.5, 97.6)	
**Median MCH levels across categories of dietary diversity score:**			
Low dietary diversity	58	31.0 pg/cell (29.5, 32.8)	0.019
Medium to high dietary diversity	73	30.0 pg/cell (28.4, 31.6)	

CRP, C-reactive protein; MCV, mean corpuscular volume; MCH, mean corpuscular haemoglobin.

No significant associations were found between dietary diversity scores and any of the blood parameters (*p* > 0.05), except for median MCV (*p* = 0.029) and median MCH (*p* = 0.019). Median MCV were within the normal range for both the group with low dietary diversity scores (94.9 fl) and those with medium to high dietary diversity scores (92.9 fl). Similarly, median MCH were also within the normal range for those who had a low dietary diversity score (31.0 pg/cell), as well as those who had a medium to a high score (30.0 pg/cell). No significant associations were found between haemoglobin and the intake of flesh meats and fish food group (*p* = 0.511) and the organ meats group (*p* = 0.281).

## Discussion

With this study, we aimed to assess the dietary diversity, presence of anaemia and related factors (menstruation and contraceptive use) in a group of women aged 25–49 years living in low socio-economic townships in the rural Free State towns of Trompsburg, Philippolis and Springfontein. As a result of the significant effects of anaemia on human health (e.g. reduced resistance to blood loss during delivery leading to increased maternal morbidity and mortality) (Kassebaum & GBD 2016), it was considered desirable to determine the need for intervention studies within this population.

Almost half of the women in this study had a low dietary diversity score, indicating that their diet needed attention in terms of the nutrient adequacy. The prevalence of low dietary diversity in this study was similar to that of the SANHANES-1 study (Table 3) (Shisana et al. 2013).

Almost a quarter of the women did not consume flesh meat and fish, which are excellent sources of the more bioavailable haem iron. The diets of the women seemed to be mostly starch-based, with low intakes of other iron-rich foods including organ meats, eggs and dark green leafy vegetables. Only a quarter of the women consumed dark green leafy vegetables, and an even smaller proportion ate legumes, which are also a rich source of folate. Dark green leafy vegetables are excellent sources of folate and the less bioavailable form of non-haem iron (Milman 2011), the absorption of which can be impaired by phytates present in some grains. The low intake of iron and folate-rich sources was similar to findings in a study conducted on non-pregnant women older than 19 years living in rural areas in KwaZulu-Natal (Kolahdooz, Spearing & Sharma 2013).

In this study, only a small proportion of the women had low haemoglobin levels indicative of anaemia. This is much lower than that reported in the SANHANES-1 study, where almost a quarter of the women between 16–35 years and 35–44 years in South Africa and 17.6% of women between 16 and 35 years in the Free State suffered from anaemia (Shisana et al. 2013). The prevalence of anaemia in this study was also much lower than the results from a systematic review by Stevens et al. (2013). Their review included population representative data where, in 2011, 29% of non-pregnant South African women aged 15–49 years suffered from anaemia. According to the National Food Consumption Survey – Fortification Baseline (NFCS-FB) survey, conducted in 2005 among women of reproductive age (15–35 years), 23.2% of women in the Free State Province suffered from anaemia (Labadarios et al. 2007). These studies, however, did not report on menstruation and contraceptive use, which may account for some of the differences.

In our study population, almost half of the women were not menstruating, and a large proportion of the sample made use of injectable contraceptives. Both of these factors may have decreased their risk of developing iron deficiency and thus possibly contributed to the low prevalence of iron deficiency in the current sample. Indeed, women in the sample who were still regularly menstruating had significantly lower median haemoglobin levels than those who were not. Results from the current study showed that a large number of women were currently or had previously made use of injectable contraceptives. Studies by Bellizzi and Ali (2018) and Haile et al. (2017) support the fact that the risk for anaemia is decreased with the use of contraceptives. The SANHANES-1 study did not report on contraceptive use and the effects thereof on anaemia.

Iron deficiency anaemia develops in three stages. The first stage is characterised by the depletion of iron stores, which is confirmed by low serum ferritin levels. During the second stage, serum ferritin decreases further with transferrin saturation decreasing as well. In the third stage, haemoglobin levels decrease, microcytosis and hypochromia of the erythrocytes develop, and signs and symptoms of anaemia appear (Gaw et al. 2008).

Low MCV levels, indicating microcytic anaemia, and low MCH levels, indicating hypochromic anaemia, in combination with decreased haemoglobin levels indicate iron deficiency anaemia. A quarter (25.2%) of the women in our study had increased levels of MCH, which is seen in macrocytic anaemia. Considering these factors, in combination with ferritin levels, it is evident that a very small percentage (1.4%, 1/134) had low iron stores. Serum ferritin levels in this study were much higher than reported in SANHANES-1, where 13.5% of women aged 16–35 years had low serum ferritin levels (Shisana et al. 2013). The higher serum ferritin levels in this study may have been linked to contraception, as a study on non-pregnant women, aged 18–40 years, in Bangladesh, Chile, China, the Dominican Republic, Pakistan, Thailand and Tunisia, in the period 1988–1992, concluded that contraceptive use has a beneficial effect on ferritin levels (UNDP/UNFPA/WHO/WB 1998).

It is important to consider that serum ferritin levels may be elevated because of inflammation and infection (Gaw et al. 2008). As ferritin is an acute phase protein that is sensitive to inflammation, some authors suggest that ferritin may rather be a marker of inflammation than iron status in individuals who are suffering from chronic inflammation, commonly seen in overweight and obese individuals (Dignass, Farrag & Stein 2018; Khan et al. 2016). In order to correct ferritin for inflammation, Thurnham, Northrop-Clewes and Knowles (2015) proposed a method using CRP and α1-acid glycoprotein (AGP). AGP was unfortunately not measured as part of the main study and adjusted ferritin could therefore not be determined. It is, however, important to note that almost a third of the women in this study presented with elevated ferritin levels. Considering that CRP was elevated in almost half (45.0%) of the women, and a significant association was found between elevated CRP and ferritin levels, prevalence of iron deficiency could thus be underestimated. Cappellini et al. (2017), proposed that iron deficiency be diagnosed in the presence of inflammatory conditions when serum ferritin levels fall below 100 mcg/L. When applying this cut-off value to the women in this study who presented with inflammation (elevated CRP), 74.1% (20/27) suffered from iron deficiency. Although this method has only recently been proposed, it is worth noting that the prevalence of iron deficiency can be greatly affected by the presence of inflammation.

When taking into account the WHO (2007) haemoglobin and serum ferritin cut-off values for diagnosing iron status, one woman (0.7%) suffered from iron deficiency anaemia (low ferritin and haemoglobin) and two women (1.5%) had an iron deficiency (low ferritin only). Iron deficiency anaemia was present in 9.7% of women of reproductive age in the SANHANES-1 study. Differences in the prevalence of anaemia and iron deficiency anaemia could also be attributed to the older median age of the women in this study compared to the SANHANES-1 study (Shisana et al. 2013). Women of younger fertile age have a greater risk for developing anaemia, particularly because of iron deficiency, because of blood loss during menstruation (Yeasmin et al. 2010).

Homocysteine levels rise and spill into the circulation when either folate or vitamin B12 is lacking, thus possibly indicating folate or vitamin B12 deficiency (Litchford 2012). Overall, 3.8% of the women had red cell folate levels below the reference value, indicating low folate status, which was much lower than the estimated prevalence of folate deficiency in 25% – 72% of women of reproductive age (Milman 2011). This difference could be because of the small sample size in this study, but also because of the intake of starchy staples, fortified with folic acid, by all of the women. Optimal folate status in women of childbearing age is of utmost importance as neural tube closure occurs at 28 weeks of pregnancy, a time when most pregnant women do not yet know that they are pregnant (Derbyshire 2011).

Median MCV and MCH decreased significantly as dietary diversity scores increased; however, all of these medians were still within the normal range. The unexpected direction of these associations may reflect on the effects of the mandatory fortification of food in South Africa. Studies conducted by Keats et al. (2019) and Garcia-Casal et al. (2018) found that food fortification had a small, but significant effect on the lowering of the prevalence of anaemia.

Although no significant association could be found between any of the blood parameters, except for MVC and MCH, and dietary diversity score, as well as any of the blood parameters and the flesh meats and fish food group and the organ meats group, there is literature suggestive of the fundamental importance of iron and folate in maternal health (WHO 2006). When considering what is known now regarding the low prevalence of anaemia and the adequacy of the women’s diets in this study, it may possibly be an indication of the effect of the mandatory food fortification of maize meal and wheat flour with folic acid and iron, amongst others, that came into effect in South Africa in October 2003 (DoHSA 2007). Food fortification may be a solution to address micronutrient malnutrition, particularly in terms of iron and folate; however, implementing other solutions as well as further studies may add value.

## Limitations of the study

The small sample size proved to be the greatest limitation of this study, which may question whether it is representative. The older median age may also have impacted on the representativity as the majority of the women may have presented with menopause. This could have impacted on the low prevalence of anaemia. The prevalence of iron deficiency and iron deficiency anaemia may also have been underestimated as it was not possible to adjust for inflammation in this study. However, we believe that this study is still of value as it provides an indication of the dietary adequacy and the prevalence of anaemia in these women.

The dietary assessment reflects only one day of the diet of the women, which is not necessarily an accurate representation of the overall dietary adequacy. When interpreting dietary diversity scores, it is important to keep in mind that the quantity of food consumed is not measured; the diet can vary across seasons where some foods are only available in large quantities and at low affordable prices for short periods of time; and that the variety available in rural areas may differ from that of urban areas (FAO 2011).

## Conclusion and recommendations

The women in this study consumed a diet with moderate variety which was based on starchy staple foods. Even though these women consumed little foods from the iron and folate containing food groups, the prevalence of iron deficiency, iron deficiency anaemia and folate deficiency was low. The low prevalence could be attributed to the large percentage with elevated CRP levels, almost half of the women not menstruating and the older median age.

The results of this study will be used to plan sustainable community-based intervention strategies to improve the dietary adequacy of the women living in the Free State, where the dietetics students from the Department of Nutrition and Dietetics of the University of the Free State provide key nutrition services. These strategies should be appropriate and culturally acceptable and should involve the diversification of the diets of women living in the Free State, specifically focusing on improving the intake of iron and folate-rich food sources, as well as reducing those factors that may inhibit the absorption. We believe that projects encouraging the growing of vegetable gardens that are currently being implemented in the low socio-economic communities in Trompsburg, Philippolis and Springfontein should receive more attention, along with the promotion of raising chickens.

These interventions are important, even though the prevalence of anaemia in our study population is low, as the consequences of the nutrition transition may still impact on the health of these women and their offspring. We believe that the effectiveness of already established nutrition intervention strategies, as well as the strategies mentioned earlier, would benefit from further research on this population. Also, a follow-up study to identify the reasons for high levels of serum ferritin is recommended.

Reducing the burden of disease in developing communities in South Africa can be expected to contribute to sustainable livelihoods and improved health outcomes.
